# Painful Enlarging Cervical Mass in Young Male

**DOI:** 10.5811/cpcem.6664

**Published:** 2024-07-31

**Authors:** Jacob Lawing, Jeremy Towns, Matthew A. Heimann

**Affiliations:** University of Alabama at Birmingham, Department of Emergency Medicine, Birmingham, Alabama

**Keywords:** *scrofula*, *neck mass*, *tuberculosis*, *lymphadenitis*

## Abstract

**Case Presentation:**

A 32-year-old male who recently immigrated from Kenya presented to the emergency department (ED) with a painful, enlarging, right-sided neck mass for eight weeks duration. Point-of-care ultrasound was used to reveal a large cystic mass with internal septations and numerous hypoechoic round lesions. Initial tuberculosis blood test ordered in the ED was positive with cultures ultimately growing *Mycobaceterium tuberculosis*.

**Discussion:**

Scrofula should be considered in the differential in patients presenting with enlarging neck masses who have epidemiological risk factors for tuberculosis.

Images in EM Capsule What do we already know about this clinical entity?

*Scrofula, or mycobacterial lymphadenitis, is a type of extrapulmonary tuberculosis that commonly presents as an enlarging mass within the cervical or supraclavicular lymph nodes.*

What is the major impact of the images?

*Identification of a cystic mass with internal septations on point-of-care ultrasound (POCUS) can help distinguish this entity from other causes of cervical masses.*

How might this improve emergency medicine practice?

*The use of POCUS on cervical masses in patients with endemic risk factors can greatly aid in the diagnosis of scrofula.*

## CASE PRESENTATION

**Image 1.**
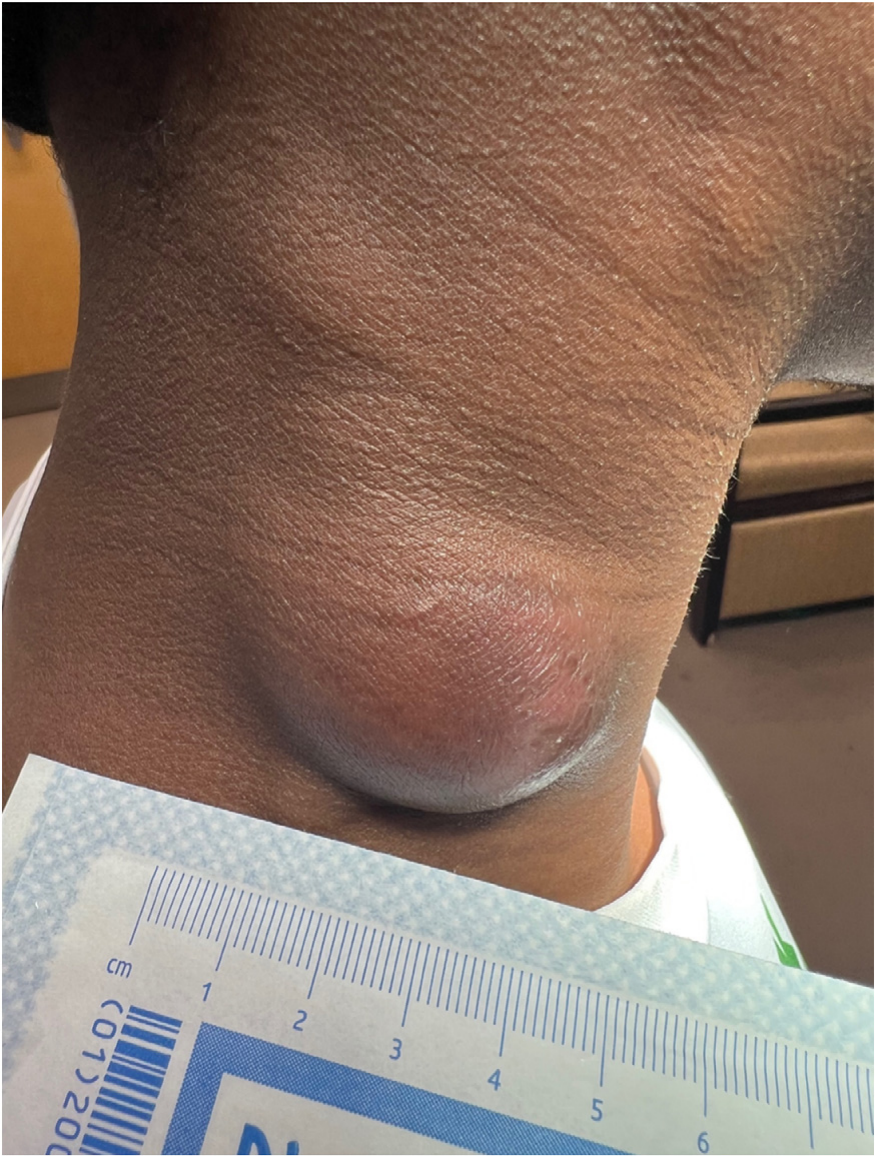
Right-sided fluctuant cervical mass with overlying erythema.

A 32-year-old male presented to the emergency department (ED) due to a painful, enlarging, right-sided neck mass for eight weeks. Notably, he had immigrated from Kenya approximately one year prior. He reported subjective fevers but denied weight loss, night sweats, or pulmonary symptoms. Physical exam revealed a moderate-sized area of focal swelling noted to right lateral neck associated with cervical lymphadenopathy (Image 1).

**Image 2.**
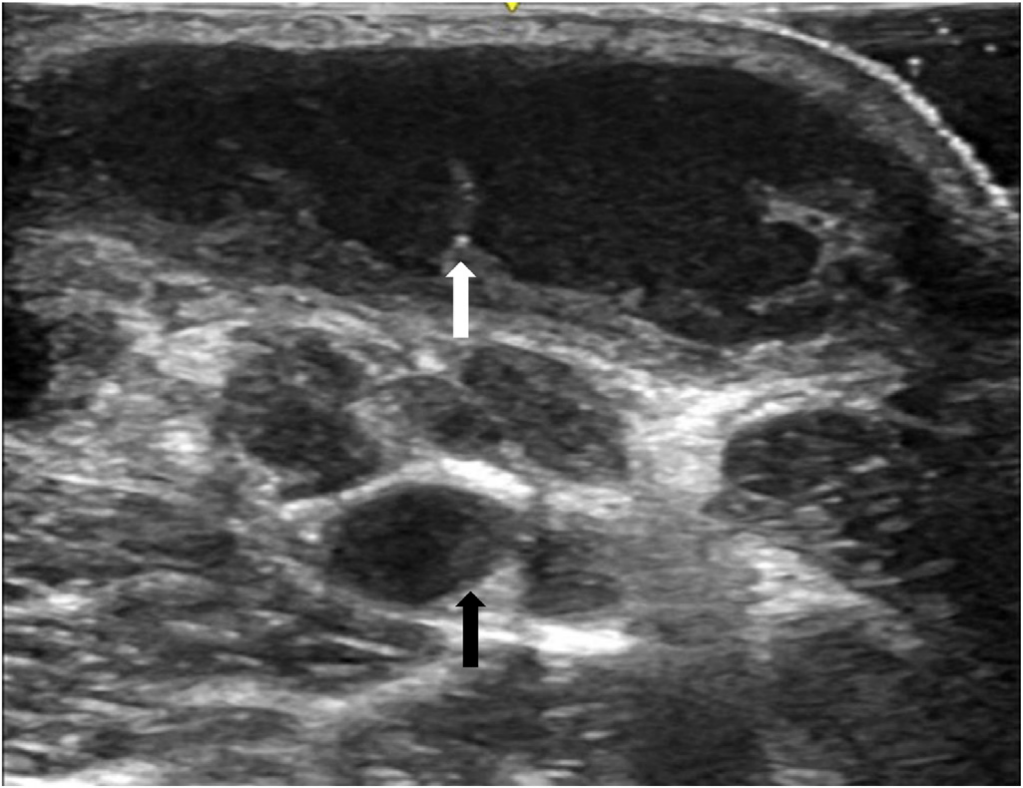
Point-of-care ultrasound revealing large cystic mass with internal septations (white arrow) and cervical adenopathy (black arrow).

**Image 3.**
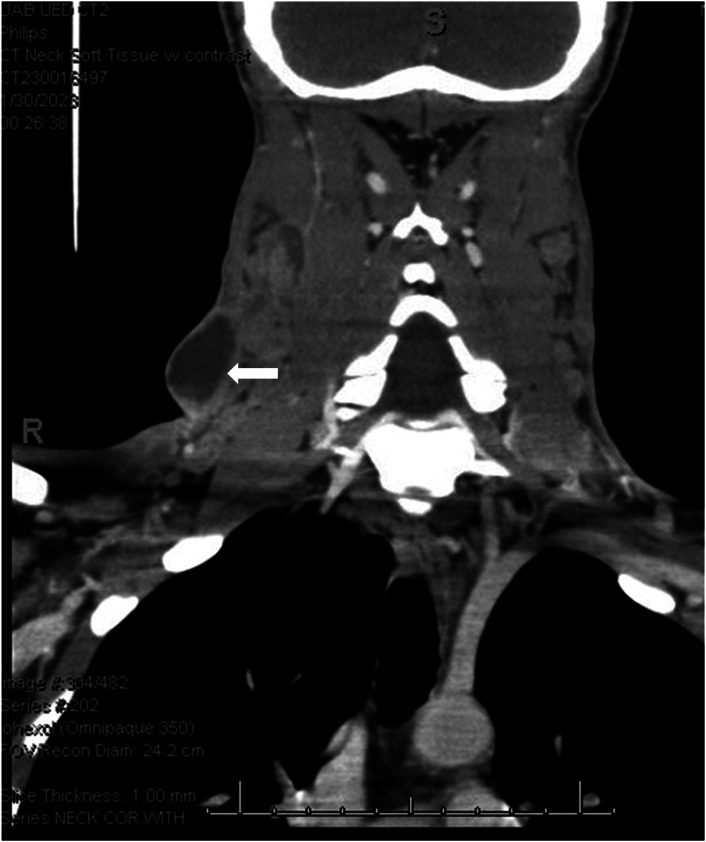
Computed tomography of the neck with intravenous contrast revealed multiple necrotic and cystic-appearing lymph nodes (arrow).

A T-SPOT.TB test (an interferon-gamma release assay) was ordered from the ED and returned positive. Empiric treatment with cefepime was started, and the patient was admitted to the hospital for ultrasound-guided lymph node biopsy. Limited point-of-care ultrasound (Image 2) and computed tomography of the neck with contrast (Image 3) were performed.

## DISCUSSION

This diagnosis of scrofula was ultimately made through acid-fast bacillus culture, which grew *Mycobacterium tuberculosis.* Scrofula, or mycobacterial lymphadenitis, is a type of extrapulmonary tuberculosis (TB) caused by hematogenous or lymphatic dissemination of pulmonary TB or reactivation of latent TB. Common presentations include an enlarging mass with or without tenderness and erythema located within the cervical or supraclavicular lymph nodes.^1^ Associated symptoms include fever, rigors, and night sweats.^2^ Point-of-care ultrasound of tuberculous nodes will be hypoechoic and round, with intranodal cystic necrosis and adjacent soft-tissue edema. Diagnosis is made by histopathology along with a smear of acid-fast bacilli and culture of lymph nodes. Treatment includes rifampicin, isoniazid, pyrazinamide, and ethambutol. It is imperative to obtain appropriate imaging such as ultrasound or computed tomography in enlarging neck masses with epidemiologic risk factors of TB and ensure close follow-up.
